# Falciform technique in laparoscopic revision of distal catheter obstruction: an evaluation

**DOI:** 10.1097/MS9.0000000000002066

**Published:** 2024-04-17

**Authors:** Trong Yen Nguyen, Quang Dung Tran, Van Hiep Pham

**Affiliations:** a Department of Neurosurgery; b Department of Digestive Surgery, 108 Military Central Hospital, Vietnam

**Keywords:** falciform ligament, hydrocephalus, laparoscopic, ventriculoperitoneal shunt (VPS)

## Abstract

**Background:**

Ventriculoperitoneal (VP) shunting is a cornerstone treatment for hydrocephalus, a condition characterized by the abnormal accumulation of cerebrospinal fluid in the ventricles of the brain. Despite its efficacy, this procedure is associated with various complications, among which distal catheter obstruction poses significant challenges. This study aimed to evaluate the effectiveness of the *‘falciform technique’* in laparoscopic revision of distal catheter obstructions, offering a novel approach to mitigate this prevalent issue.

**Materials and methods:**

This study retrospectively analyzed 28 patients with VP shunt distal catheter obstructions who underwent laparoscopy-assisted shunt revision between January 2016 and June 2022. All of these were done using the *‘falciform technique’* with the fixation of the distal catheter to the falciform ligament in supra-hepatic space.

**Results:**

The most common etiology of primary shunt surgery was hydrocephalus, followed by intracranial hemorrhage (42.9%) and traumatic brain injury (32.1%). Normal pressure hydrocephalus occurs in 14.3% of cases. Fifteen patients (53.6%) required revision surgery within 1 year of index surgery. Thirteen patients (46.4%) underwent revision surgery more than 1 year after the index surgery, either as a first revision or subsequent revision. The average surgery time was 32.1±14.7 min and hospital stay was 4.2±1.8 days. After a mean follow-up period of at 20.3±8.7 months, except for three patients who died from other causes (two patients due to pneumonia and one due to exhaustion), there were no shunt-related complications in the remaining 25 patients.

**Conclusions:**

Laparoscopy with the application of *‘falciform technique’* is a safe and highly effective method in distal catheter obstruction revision following VP shunt.

## Introduction

Highlights

- Evaluation of the effectiveness of the *‘Falciform Technique’* in laparoscopic revision of distal catheter obstruction following ventriculoperitoneal shunting.
- A retrospective study of 28 patients, highlighting the reduction of shunt-related complications using this technique.
- Demonstrates the falciform technique as a safe and effective method in managing distal catheter obstructions in ventriculoperitoneal shunts.

Hydrocephalus affects ~175 per 100 000 adults worldwide^1^. Ventriculoperitoneal (VP) shunt insertion remains the mainstay treatment for hydrocephalus^2^. Although shunt placement is a common and safe procedure, several complications may occur. The incidence of shunt malfunction after initial placement occurs in ~25–35% of patients after one year. Up to 24% of VP shunt patients experience shunt-related complications, and the type of dysfunction may vary depending on the age of the bypass^3^.

Shunt catheter obstruction is by far the most common cause of shunt malfunction, yet the factors that contribute to this problem remain elusive. Obstruction can occur in the proximal catheter, valve, or distal catheter. With the advent of laparoscopic techniques, distal catheter obstructions can be diagnosed and managed laparoscopically. Laparoscopy-assisted shunt revision in selected cases might be a less invasive and more effective option for pediatric patients with multiple intra-abdominal manipulations because the laparoscopic approach allows doctors to perform better catheter positioning, lysis of fibrotic bundles, and peritoneal inspection, without any additional complications^4^. The adult literature has indicated that laparoscopic VP shunt revision offers lower rates of failure, particularly with reference to certain subsets of etiology, such as normal-pressure hydrocephalus^5^.

More studies have revealed that the hepatic septal space, which is the highest position of the abdominal cavity in both the supine and sitting positions, is ideal for the abdominal shunt to prevent distal catheter obstruction. Shao *et al*.^6^ explored a novel fixation method in the laparoscopically assisted VP shunt with use of the liver falciform ligament as a natural support for fixation of the distal shunt catheter, which called the *‘falciform technique’*. The application of *‘falciform technique’* via laparoscopy has been proven to be effective in reducing the complication rate of distal obstruction^7^. However, there are few articles mentioning the use of this technique in distal catheter obstruction revision. The aim of this study was to evaluate the effectiveness and safety of the application of *‘falciform technique’* via laparoscopy in distal catheter obstruction revision.

## Patients and methods

We report a retrospective study including 28 patients with distal catheter obstruction secondary to VP shunt for hydrocephalus, treated laparoscopically, between January 2016 and June 2022. All patients had a history of VP shunt insertion and presented with clinical manifestations of shunt obstruction. Clinical evaluation of the shunt system revealed distal resistance with manual valve compression, and computed tomography revealed dilated ventricles. For all patients, radiography was performed to exclude the disconnected system, whereas abdominal ultrasonography was performed to diagnose any possible cause of obstruction. Shunt infections were excluded in all patients through blood and cerebrospinal fluid (CSF) tests. This study was conducted in accordance with the strengthening the reporting of cohort, cross-sectional, and case–control studies in surgery (STROCSS) criteria^8^.

### Surgical procedure

**Figure 1:**
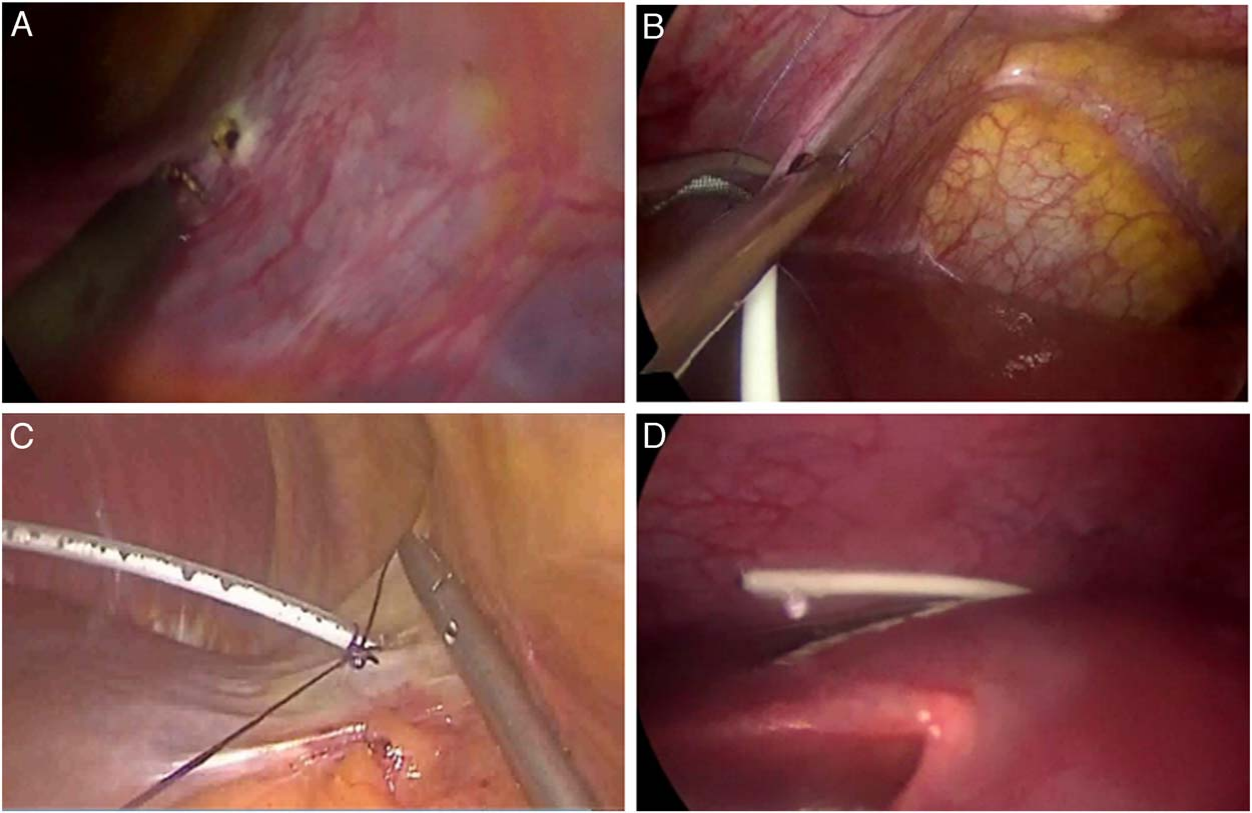
Endoscopic views showing the Falciform Technique. (A) Creation of a hole in the falciform ligament using electrocautery. (B) Insertion of the trimmed distal catheter through the hole. (C) Fixation of the catheter with a suture. (D) Checking catheter patency after placement over the liver dome.

**Figure 2:**
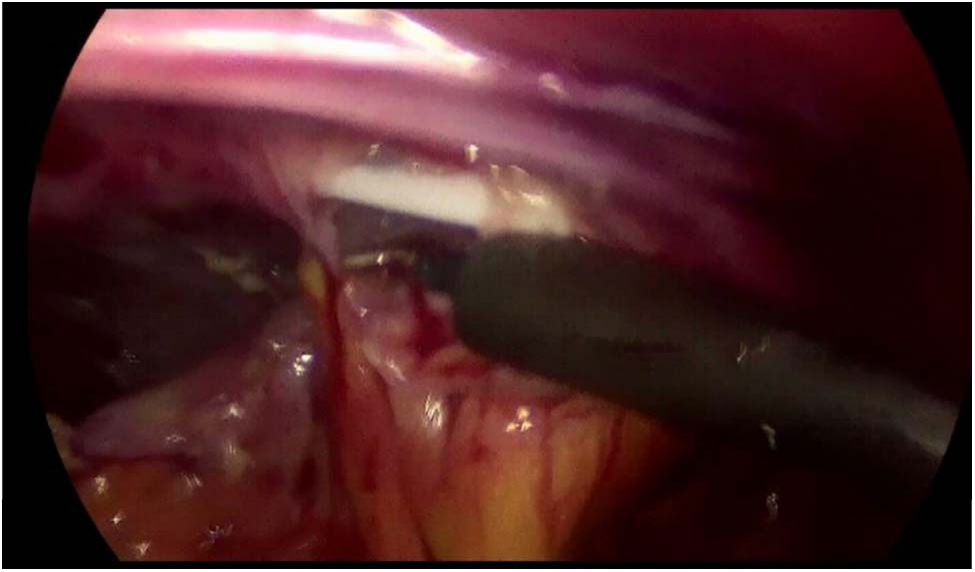
Releasing distal tip of the shunt entrapped in peritoneal adhesions, and for performing adhesiolysis.

The surgical procedures were performed under general anesthesia. In the present study, a team of neurosurgeons and general surgeons participated in determining surgical approaches and performing surgical procedures. A laparoscopic procedure was planned to release the distal tip of the shunt entrapped in peritoneal adhesions and to perform adhesiolysis and repositioning of the shunt tube (Fig. 1). The procedure starts with a small supra umbilical incision for the creation of pneumoperitoneum (pressure of 14 mmHg) using a Verrus needle (closed technique). The laparoscope was inserted through the supra umbilical incision via a 10 mm trocar and connected to the video system. Another 5 mm trocar was placed in the upper-left quadrant. Diagnostic laparoscopy was then performed, with lysis of adhesions, if necessary (Fig. 2). A previously inserted distal catheter was then inserted and assessed. If the catheter was clogged by a clot, it was removed. All existing catheters were retained and not replaced with a new catheter.

A small hole was created in the nonvascular part of the falciform ligament using electrocautery (Fig. 1A). The distal catheter was trimmed to a length of no more than 30 cm within the abdominal cavity, and tested for patency under direct vision. The distal catheter was placed through the hole in the falciform ligament from the left side to the right side of the falciform ligament (Fig. 1B) and fixed using a suture (Fig. 1C). The distal catheter was placed through the falciform defect and draped over the dome of the liver in the subdiaphragmatic space so that the distal end of the catheter would reach the hepatic flexure to drain CSF directly into the right paracolic gutter. Catheter patency for CSF drainage was examined again by pressing the proximal valve (Fig. 1D). The abdomen was then desufflated, and the port sites were closed with subcuticular sutures.

## Results

### Patient demographics

The patient demographics are presented in Table 1. The study group comprised of 12 males and 16 females. The patients’ ages ranged from 41 to 69 years, with a mean age of 52.5±14.1 years. The most common etiology of primary shunt surgery was hydrocephalus, followed by intracranial hemorrhage (42.9%) and traumatic brain injury (32.1%). Normal pressure hydrocephalus occurs in 14.3% of cases. Fifteen patients (53.6%) required revision surgery within 1 year of index surgery. Thirteen patients (46.4%) underwent revision surgery more than 1 year after the index surgery, either as a first revision or subsequent revision. However, in the previous revision, assisted laparoscopy was not used.

**Table 1:** Patient demographics.

Age (years)	52.5±14.1 (41–69)
Sex	
Male (*n*, %)	12 (42.9)
Female (*n*, %)	16 (57.1)
BMI (kg/m^2^)	19.5±4.9
Hydrocephalus etiology	
Post-ICH (*n*, %)	12 (42.9)
Post-TBI (*n*, %)	9 (32.1)
NPH (*n*, %)	4 (14.3)
Meningitis (*n*, %)	3 (10.7)
Patients with shunt revisions	
Patients with 1st revision within 1 year (*n*, %)	15 (53.6)
Patients with revision >1 years (*n*, %)	13 (46.4)

ICH, intracranial hemorrhage; NPH, normal pressure hydrocephalus; TBI, traumatic brain injury.

Intraoperative findings revealed peritoneal adhesions in 57.1% of cases and pseudocysts in 32.1%, while clots were found in three cases (10.7%). The outcomes after revision surgeries utilizing the ‘Falciform Technique’ via laparoscopy are presented in Table 2. The average surgery time was 32.1±14.7 min and hospital stay was 4.2±1.8 days. Except for three patients who died from other causes (two patients due to pneumonia and one due to exhaustion), there were no migrations of the peritoneal shunt catheters, and no distal obstructions or infection in any of the 25 patients after the average follow-up period of 20.3±8.7 months. None of the patients reported perforation, pseudocyst formation or other complications that could be attributed to the surgery.

**Table 2:** Outcomes after revision surgeries with of ‘falciform technique’ via laparoscopy.

Causes of distal obstruction	
Clot (*n*, %)	3 (10.7)
Peritoneal adhesion (*n*, %)	16 (57.1)
Pseudocyst (*n*, %)	9 (32.1)
Surgery duration, mean (mins)	32.1±14.7
In-hospital (days)	4.2±1.8
Follow-up (months)	20.3±8.7
Results	
Death (*n*, %)	3 (10.7)
Shunt dysfunction (*n*, %)	0

## Discussion

CSF shunting was designed more than 50 years ago to address an apparently simple problem (i.e. hydrocephalus) by transferring excess CSF from the brain to another bodily region capable of reabsorbing it. In recent decades, the preferred site for CSF reabsorption has been the peritoneum, making VP shunt with adjustable valves the most frequently used shunt worldwide for both pediatric and adult hydrocephalus^9,10^. VP shunts are generally considered a simple procedure with a low perioperative risk profile; however, malfunction rates remain high, with ~40% of pediatric shunts and 29% of adult shunts failing in the first year of placement and 45 to 81% of patients with a shunt requiring one surgical shunt revision in their lifetime^10^. In children, proximal ventricular shunt obstruction/malfunction is more common, while in adults, most VP shunt malfunctions are related to the distal peritoneal shunt^11^. Broggi *et al*. proposed a practical, rapid, and safe algorithm for testing the VP shunt function. The proposed method has the advantage of being able to identify the exact point of VP shunt malfunction/obstruction/failure (proximal catheter, valve device, or distal catheter), thus allowing, in most cases, a selective system revision to be performed, reducing the invasiveness and complication rate of the redo procedure^12^.

In a retrospective study of 810 cases, Naftel *et al*.^13^ concluded that blind placement of catheters in open VP shunts might cause mistakes and increase the incidence of distal catheter obstruction (35.7%), which was reduced by assisted laparoscopy (4.8%). A systematic review and meta-analysis by Phan *et al*.^14^ demonstrated that the laparoscopic technique in VP shunt surgery is associated with reduced shunt failure and abdominal malposition compared to the open laparotomy technique, with no significant difference in the rates of infection or other complications.

Kim *et al*.^15^ first described laparoscopic management of abdominal complication. In revision surgery for abdominal complications after VP shunt visualization of the whole abdominal cavity is fundamental to properly address the problem, and the laparoscopic approach is valuable because it is safe, fast, and much less invasive than laparotomy. The laparoscopic approach allows better catheter positioning, lysis of fibrotic bundles, and peritoneal inspection without any additional complication^16^. Nfonsam *et al*. found that distal VP shunt complications could be safely and effectively managed laparoscopically. This approach allows the intra-abdominal portion of the catheter to be assessed and problems to be managed, thereby salvaging the existing shunt and avoiding the potential morbidity associated with additional VP shunt placement^17^.

For a long time, we usually revised for the peritoneal end by externalizing the tube and checking for CSF flow, which are considered diagnostic and therapeutic procedures. The rationale supporting conventional laparotomy includes factors such as the simple learning curve, as it can be performed by neurosurgeons, and its established success rate. However, this standard technique is associated with multiple complications, especially shunt revision, which is associated with a higher incidence of infection and abdominal adhesions and may lead to visceral injury; sometimes, the tube may be broken during extraction. However, this is problematic for several reasons. First, the larger incision and scarred tissues predispose the patient to surgical site infection and hernia formation. Second, visceral adhesions predictably form on the underside of the prior open incisions, making inadvertent enterotomy more likely. Third, the surgeon may unknowingly dissect the peritoneum away from the muscular abdominal wall and create a pocket considering that, the surgeon is within the peritoneal cavity itself. Shunt placement in this pocket can result in distal shunt obstruction, pseudomeningocele, or shunt migration into the subcutaneous space.

Studies have revealed that the hepatic septal space, which is the highest position of the abdominal cavity in both the supine and sitting positions, is ideal for the abdominal shunt to prevent omental wrapping and organ damage^18,19^. Svoboda *et al*.^20^ placed the catheter through a falciform ligament defect, which reduced migration and obstruction in idiopathic normal pressure hydrocephalus (iNPH). During treatment in 36 patients, Wang *et al*.^21^ placed the catheter into the right subphrenic space through a hole in the sickle ligament with no complications during follow-up. Utilizing the falciform ligament of the liver and the suprahepatic recess to suspend and maintain the shunt, outcomes may result in fewer iatrogenic intra-abdominal injuries, enhanced ease of shunt removal, a large surface area for absorption of drained CSF, and fewer adhesions secondary to device placement^22^.

## Conclusions

Through a detailed evaluation of the Falciform Technique applied in laparoscopic revision for distal catheter obstruction, our study demonstrated its high effectiveness and safety. This technique leverages the advantages of laparoscopic surgery and presents a promising method for managing this complex condition. Our findings suggest that incorporating this technique can significantly improve patient outcomes in similar clinical scenarios.

### Limitations

Although the results are promising, the retrospective nature of our study and the limited sample size warrant cautious interpretation. Future studies with larger, randomized controlled trials are necessary to further validate the efficacy of the falciform technique. Moreover, multicenter collaboration would be beneficial in enhancing the generalizability of our findings.

## Ethical approval

Ethical approval for this study was provided by the Ethical Committee NAC of 108 Military Central Hospital, Hanoi, Vietnam on 05 September 2023.

## Consent

Written informed consent was obtained from all participants. A copy of the written consent form is available for review by the Editor-in-Chief of this journal upon request.

## Sources of funding

This research received no specific grant from any funding agency in the public, commercial, or not-for-profit sectors.

## Author contribution

T.Y.N., and Q.D.T.: study concept, data collection, data analysis, and writing the paper; V.H.P.: study concept and writing the paper.

## Conflicts of interest disclosure

All authors declare no conflicts of interest.

## Research registration unique identifying number (UIN)

1. Name of the registry: www.researchregistry.com.
2. Unique identifying number or registration ID: researchregistry9930.
3. Hyperlink to your specific registration (must be publicly accessible and will be checked): https://www.researchregistry.com/browse-the-registry#home/registrationdetails/65a935f8f2f4f30028426e37/.

## Guarantor

Trong Yen. Nguyen, MD, PhD and Quang Dung Tran, MD.

## Data availability statement

Data are available, available upon reasonable request.

## Provenance and peer review

Not commissioned, externally peer reviewed.
